# Genome-wide identification, classification, and expression pattern analysis of the TCP transcription factor family in carrot

**DOI:** 10.3389/fpls.2026.1788962

**Published:** 2026-02-26

**Authors:** Jian-Hua Zhou, Qiang Wu, Li Zhang, Pei-Yan Chen, Xiao-Jing Zhang, Na Fang, Bing-You Wang, Yi-Xin Zhang, Nong-Yi Zheng, Ai-Sheng Xiong

**Affiliations:** 1 Zhengzhou Academy of Agricultural Science and Technology, Zhengzhou, China; 2 State Key Laboratory of Crop Genetics & Germplasm Enhancement and Utilization, Ministry of Agriculture and Rural Affairs Key Laboratory of Biology and Germplasm Enhancement of Horticultural Crops in East China, College of Horticulture, Nanjing Agricultural University, Nanjing, China; 3 Zhengzhou Agricultural Economic Development Center, Zhengzhou, China

**Keywords:** bioinformatics analysis, carrot, expression profiling, genome-wide analysis, root development, TCP transcription factor

## Abstract

Carrot (*Daucus carota* L.) is an important root vegetable crop in the Apiaceae family, widely cultivated worldwide, with high nutritional and economic value. The *TCP* gene family is a plant-specific transcription factor family containing an atypical basic helix-loop-helix (bHLH) structure, which plays a crucial role in regulating plant growth and development and responding to stresses. In this study, genome-wide identification and systematic analysis of the *TCP* gene family in carrot were conducted using bioinformatics methods. The results showed that a total of 50 *DcTCP* family genes were identified in the carrot genome. The molecular weights of the proteins encoded by these genes ranged from 6056 to 53,512.5 Da, most of which were hydrophilic and unstable proteins, and all were localized in the nucleus. The *DcTCP* gene family had a relatively simple structure with a small number of introns, and 48 genes contained motif 1. Cis-acting element analysis revealed that *DcTCP* genes contained elements related to light response, stress response, and hormone response, participating in various physiological regulatory pathways. Phylogenetic analysis classified them into three subfamilies: PCF, CIN, and CYC/TB1. Among them, the PCF subfamily had the most members (36), accounting for 72% of the total family. Chromosomal localization indicated that the 50 *DcTCP* genes were unevenly distributed on 9 chromosomes, and whole-genome duplication (WGD) was the main driving force for the expansion of this family. Regulatory network prediction identified 12 regulatory miRNAs with targeting relationships to *DcTCP* genes, among which *miR319* had the most target genes (7 target genes), and 28 DcTCP proteins could form 214 pairs of protein-protein interactions. Expression pattern analysis showed that *DcTCP* genes exhibited specific expression at different developmental stages of carrot roots. Some genes (such as *DcTCP33* and *DcTCP37*) were highly expressed throughout the entire developmental process, while the expression levels of other genes gradually increased with the developmental stage, suggesting their involvement in the regulation of fleshy root formation and development. This study clarified the basic characteristics and potential regulatory mechanisms of the *DcTCP* gene family in carrot, enriched the research content of the plant *TCP* gene family, and provided a theoretical basis and gene resources for subsequent analysis of carrot growth and development rules and cultivation of stress-resistant and high-quality varieties.

## 1 Introduction

Carrot (*Daucus carota* L.) is a root vegetable crop of carrot species in the Apiaceae family. It originated in Central Asia and is now widely distributed around the world (Que et al., 2019). It stores a large amount of nutrients by forming hypertrophic fleshy roots, which are the main edible parts (Wang et al., 2015). Rich in carotenoids, cellulose and other substances, it has the functions of antioxidation, anti-aging and enhancing immunity (Li et al., 2021), which has high nutritional value and economic value. Like most plants of the Parsley family, carrots are diploid, with 18 chromosomes (2n = 2x = 18). The genome size is smaller than that of celery and coriander (Iorizzo et al., 2016).

TCP (Teosinte branched1/Cycloidea/Proliferating cell factor) is a plant-specific transcription factor family, which is derived from TEOSINTE BRANCHED 1 (TB1) in maize, CYCLOIDEA (CYC) in Antirrhinum majus and Proliferating Cell Factor 1/2(PCF1/2) in rice, and contains an atypical basic helix-loop-helix (bHLH) structure (Cubas et al., 1999). Cording to the amino acid differences in the domain, it is divided into two subgroups: PCF (Class I), CYC/TB1 (Class II) and CIN (Class II). By interacting with other proteins, it regulates plant growth and development (Resentini et al., 2021), metabolism (Zhang et al., 2021), signal transduction and stress response (Xu et al., 2021) and other biological activities. At present, the research on *TCP* gene family in plants mainly focuses on model plants. There are 24 *TCP* in *Arabidopsis thaliana* (Yao et al., 2007), 24 in tomato (Parapunova et al., 2014), 32 in celery (Duan et al., 2019). The ectopic expression of TCP4 in *Arabidopsis thaliana* will lead to the decrease of flowers and the fusion of sepals (Nag et al., 2009). *TCP17* regulates the synthesis of auxin *in vivo* by coupling the light signaling pathway to control cell elongation (Zhou et al., 2017). In rice, *PCF5* and *PCF8* can significantly improve the cold tolerance of rice seedlings (Martín-Trillo and Cubas, 2009). *TCP21* negatively regulates the number of tillers and reduces yield in rice (Wang et al., 2021). TCP gene family members, especially members of the class II CIN subfamily, are generally regulated by *miR319* (Sun et al., 2017; Yang et al., 2013). In *Arabidopsis thaliana*, reducing the expression of *miR319* and thereby promoting the expression of AtTCP protein can facilitate the growth and development of *Arabidopsis thaliana* roots (Baulies et al., 2022).

Previous studies have shown that TCP transcription factor family genes play an important regulatory role from the microscopic cell level to the macroscopic phenotype, and have been widely studied in many species, such as *Arabidopsis thaliana* and rice, with limited systematic analysis in root vegetable crops. In this study, bioinformatics methods were used to identify and analyze the carrot *TCP* gene family in many aspects with the help of carrot genome data, and their physical and chemical properties, evolutionary relationships, and expression patterns were comprehensively analyzed in order to further explore the function of carrot *TCP* genes. This gap limits our understanding of the molecular mechanisms underlying fleshy root development in Apiaceae crops. Elucidating the characteristics and regulatory networks of the *DcTCP* gene family will not only enrich the evolutionary and functional studies of plant TCP genes but also provide candidate genes for genetic improvement of carrot root yield and quality.

## 2 Materials and methods

### 2.1 Identification and physicochemical properties analysis of TCP gene family members in carrot

Download the carrot genome annotation file and the total sequence file in the carrot genome database (https://plants.ensembl.org/Daucus_carota/Info/Index, genome assembly: GCA001625215.1). The Arabidopsis *TCP* gene family protein sequence was downloaded from the TAIR website (https://www.arabidopsis.org/). The protein sequences of rice *TCP* gene family were downloaded from RGAP website (https://rice.uga.edu/). The protein sequences of celery *TCP* gene family were downloaded from celery website (http://celerydb.bio2db.com). The hidden Markov model file of PF03634 was downloaded from the Pfam website (http://pfam.xfam.org/) as a reference, and the HMMER v3.3.2 search was used to search the carrot genome to obtain the carrot TCP protein sequence. Using *Arabidopsis thaliana* and rice as reference species, Blastp was used to obtain homologous sequences in carrot (E-value < 10-10), then redundant and incomplete sequences were removed, and finally obtained accurate carrot *TCP* candidate genes. The physicochemical properties of TCP protein, including amino acid number, molecular weight, isoelectric point, instability index, aliphatic index and hydrophilicity, were analyzed by Protein parameter Calc of TBtools. The subcellular localization of TCP protein was predicted by WoLF-PSORT website (https://wolfpsort.hgc.jp/).

### 2.2 Chromosomal localization and collinearity analysis of TCP gene family members in carrot

The chromosome location information was obtained from the carrot genome annotation file. The chromosome distribution map of *TCP* gene family members was drawn by TBtools, and the carrot *TCP* gene family (*DcTCPs*) were named according to its position on the chromosome. The homology analysis and collinearity analysis of carrot *TCP* gene family (*DcTCPs*) were carried out by using Advanced Circos in TBtools software (Chen et al., 2020). The ratio of non-synonymous substitution rate (Ka) to synonymous substitution rate (Ks) was calculated to evaluate the selection pressure of duplicated gene pairs.

### 2.3 Phylogenetic tree construction and structure and protein domain analysis of carrot TCP gene family members

MEGA 11 software was used for phylogenetic analysis. MUSCLE was used for sequence alignment of TCP proteins from carrot, Arabidopsis and rice. Neighbor-Joining algorithm was used to construct phylogenetic tree (Bootstrap = 1000), and the Poisson model for amino acid substitution. The resulting phylogenetic tree was visualized through the online tool iTOL (https://itol.embl.de). The carrot TCP protein sequence was submitted to the MEME online tool (https://meme-suite.org/meme/tools/meme) for conservative motif analysis (motif number = 10, motif width = 10–300 aa). The annotation information of carrot *TCP* gene was submitted to TBtools software for intron and exon analysis, and the gene structure was visualized.

### 2.4 Promoter cis-element analysis and GO function analysis of carrot TCP gene family members

The *TCP* gene promoter sequence was submitted to the PlantCare website (https://bioinformatics.psb.ugent.be/webtools/plantcare/html/) for cis-acting element analysis using the 2000 bp upstream of the gene initiation site ATG as the promoter region of the gene. GO functional enrichment analysis of TCP genes was performed using the EggNOG5.0 database (http://eggnog5.embl.de/#/app/home) and visualized using TBtools.

### 2.5 Analysis of interaction network of carrot TCP gene family members

The interaction miRNAs of carrot TCP family members were predicted by psRNATarget online software (https://www.zhaolab.org/psRNATarget/). The Expect value was set to 5, and Arabidopsis miRNAs and some carrot miRNAs were used as reference. The String online website (https://www.string-db.org/, version 11.5) was used to predict the protein interaction network of carrot TCP protein with a confidence score threshold of 0.7, and *Arabidopsis thaliana* was set as the reference species.

### 2.6 Transcript abundance analysis of carrot TCP gene family members in roots at different stages and RT-qPCR

Transcript abundance analysis during carrot root development Based on the transcriptome data (SRR 2177455), RPKM (Reads Per Kilobase per Million mapped reads) was used as an indicator to measure the transcript or gene expression level. The heat map was drawn by Multiexperiment Viewer software (https://mev.tm4.org/).

The expression levels of *DcTCP* genes in carrot root at different growth stages (30d, 60d, and 90d) were determined using Hieff qPCR SYBR Green Master Mix (Yeason, Shanghai, China). Design specific primers using Primer Premier 6.0 software (**Supplementary Table S1**), *DcActin* was used as the reference gene, and its expression stability was verified using geNorm and NormFinder algorithms (Wang et al., 2016). The total reaction volume for RT-qPCR was 20 µL, which included 10 µL of SYBR Premix Ex Taq, 2 µL of cDNA template, 7.2 µL of ddH2O, and 0.4 µL of each forward and reverse primer. The thermal cycling program was as follows: initial denaturation at 95°C for 5 min, followed by 40 cycles of denaturation at 95°C for 10 s and annealing at 60°C for 30 s. The relative expression levels of the genes were calculated using the 2^−ΔΔCT^ method (Pfaffl, 2001). Each sample was performed for three biological replicates. Statistical analysis was performed using SPSS 25.0 software, and significant differences were determined by Duncan’s multiple range test at *p* < 0.05.

## 3 Results

### 3.1 Identification and classification of carrot TCP gene family

A total of 50 *TCP* gene family members were identified in the carrot genome and designated as *DcTCP1* to *DcTCP50* in sequence according to their distribution on chromosomes (**Table 1**; **Supplementary Table S2**). The 50 identified *DcTCP* family genes encode proteins with amino acid lengths ranging from 55 to 496 residues. Their molecular weights span 6056 to 53,512.5 Da, with an average of 28,857.83 Da. The theoretical isoelectric points (*pI*) of these proteins ranged from 4.73 to 9.8, and their instability indices varied from 33.81 to 65.9—with 90% of the proteins classified as unstable. The aliphatic indices of the DcTCP proteins fall between 47.38 and 83.43, and all proteins exhibited a grand average of hydropathicity (GRAVY) value less than 0, indicating they were hydrophilic. Subcellular localization prediction revealed that all *DcTCP* family members were localized in the nucleus.

**Table 1:** Information of DcTCP gene family member and physicochemical properties of the proteins in carrot.

Sequence ID	Gene ID	Number of Amino Acid	Molecular Weight	Theoretical pI	Instability Index	Aliphatic Index	Grand Average of Hydropathicity	Sub-cellular localization
KZM83458	*DcTCP49*	371	40121.43	6.77	65.24	60.27	-0.633	Nucler
KZM83459	*DcTCP50*	371	40121.43	6.77	65.24	60.27	-0.633	Nucler
KZM84511	*DcTCP46*	188	19998.6	5.9	55.77	79.89	-0.233	Nucler
KZM85695	*DcTCP47*	234	24995.13	9.45	51.49	71.84	-0.453	Nucler
KZM85865	*DcTCP48*	370	40942.38	8.84	46.65	61.43	-0.788	Nucler
KZM86556	*DcTCP39*	336	37595.56	9.14	36.08	56.99	-0.904	Nucler
KZM87518	*DcTCP40*	345	37255.93	4.89	62.72	70.06	-0.279	Nucler
KZM87519	*DcTCP41*	344	37182.84	4.73	62.04	75.06	-0.209	Nucler
KZM87523	*DcTCP42*	277	29827.11	9.01	55.55	70.43	-0.569	Nucler
KZM88277	*DcTCP43*	423	45112.9	6.31	56.97	57.71	-0.651	Nucler
KZM88362	*DcTCP44*	314	35017.68	7.07	54.69	61.18	-0.823	Nucler
KZM89192	*DcTCP45*	330	36871.11	6.68	51.4	78.33	-0.551	Nucler
KZM89456	*DcTCP35*	335	37179.77	8.99	43.69	67.85	-0.65	Nucler
KZM89519	*DcTCP36*	374	41768.35	7.85	59.18	62.54	-0.743	Nucler
KZM91111	*DcTCP37*	385	41344.47	6.76	59.88	55.79	-0.657	Nucler
KZM91355	*DcTCP38*	306	33932.53	6.4	53.14	59.02	-0.761	Nucler
KZM93295	*DcTCP33*	388	41336.49	6.53	60.38	55.85	-0.612	Nucler
KZM96345	*DcTCP34*	388	42655.64	6.17	62.82	55.62	-0.804	Nucler
KZM97506	*DcTCP30*	346	39085.71	9.18	48.97	63.96	-0.792	Nucler
KZM98143	*DcTCP31*	253	26513.38	8.69	48.12	64.11	-0.591	Nucler
KZM99649	*DcTCP32*	181	19441.48	8.51	39.98	83.43	-0.129	Nucler
KZN00654	*DcTCP25*	312	33090.37	5.11	65.9	71.89	-0.266	Nucler
KZN01014	*DcTCP26*	303	34082.46	5.92	56.58	54.42	-0.89	Nucler
KZN01084	*DcTCP27*	175	18474.97	8.9	40.23	70.29	-0.396	Nucler
KZN02218	*DcTCP28*	226	26065.55	9.8	55.52	71.73	-0.755	Nucler
KZN02590	*DcTCP29*	359	40542.11	6.28	51.98	63.87	-0.801	Nucler
KZN04402	*DcTCP17*	400	45246.91	5.96	53.53	66.12	-0.776	Nucler
KZN05846	*DcTCP18*	496	53512.5	6.41	52.97	47.38	-0.869	Nucler
KZN06062	*DcTCP19*	303	32512.58	8.51	50.74	68.32	-0.526	Nucler
KZN07043	*DcTCP20*	449	49432.16	7.13	61	54.32	-0.884	Nucler
KZN07554	*DcTCP21*	182	19428.16	8.69	53.97	76.7	-0.4	Nucler
KZN07555	*DcTCP22*	182	19445.25	9.04	52.45	77.8	-0.355	Nucler
KZN07556	*DcTCP23*	182	19445.25	9.04	52.45	77.8	-0.355	Nucler
KZN07558	*DcTCP24*	172	18675.89	6.98	55.32	70.41	-0.622	Nucler
KZN08355	*DcTCP1*	154	16493.79	9.42	50.14	55.78	-0.719	Nucler
KZN08356	*DcTCP2*	154	16493.79	9.42	50.14	55.78	-0.719	Nucler
KZN08357	*DcTCP3*	180	19002.41	6.75	41.67	67.22	-0.526	Nucler
KZN08358	*DcTCP4*	153	15877	8.86	33.81	70.85	-0.337	Nucler
KZN08359	*DcTCP5*	154	16276.46	9.23	42.77	55.78	-0.732	Nucler
KZN08360	*DcTCP6*	207	21979.83	5.19	52.41	57	-0.448	Nucler
KZN08361	*DcTCP7*	176	18585.06	7.71	38.7	66.59	-0.397	Nucler
KZN08363	*DcTCP8*	156	16805.78	6.31	48.99	59.42	-0.68	Nucler
KZN08366	*DcTCP9*	142	14939.03	9.17	52.3	61.13	-0.557	Nucler
KZN08367	*DcTCP10*	191	20249.89	5.78	48.35	66.39	-0.432	Nucler
KZN08368	*DcTCP11*	253	26801.9	5.81	45.45	69.8	-0.536	Nucler
KZN08369	*DcTCP12*	183	19248.73	6.16	43.65	69.29	-0.417	Nucler
KZN08370	*DcTCP13*	142	14951.08	9.36	45	60.49	-0.563	Nucler
KZN08371	*DcTCP14*	55	6056.83	9.77	51.24	72.73	-0.813	Nucler
KZN08388	*DcTCP15*	136	14223.11	5.01	34.33	73.97	-0.2	Nucler
KZN09938	*DcTCP16*	247	26652.84	9.37	53.91	67.98	-0.619	Nucler

### 3.2 Chromosomal localization and collinearity analysis of carrot TCP gene family

**Figure 1:**
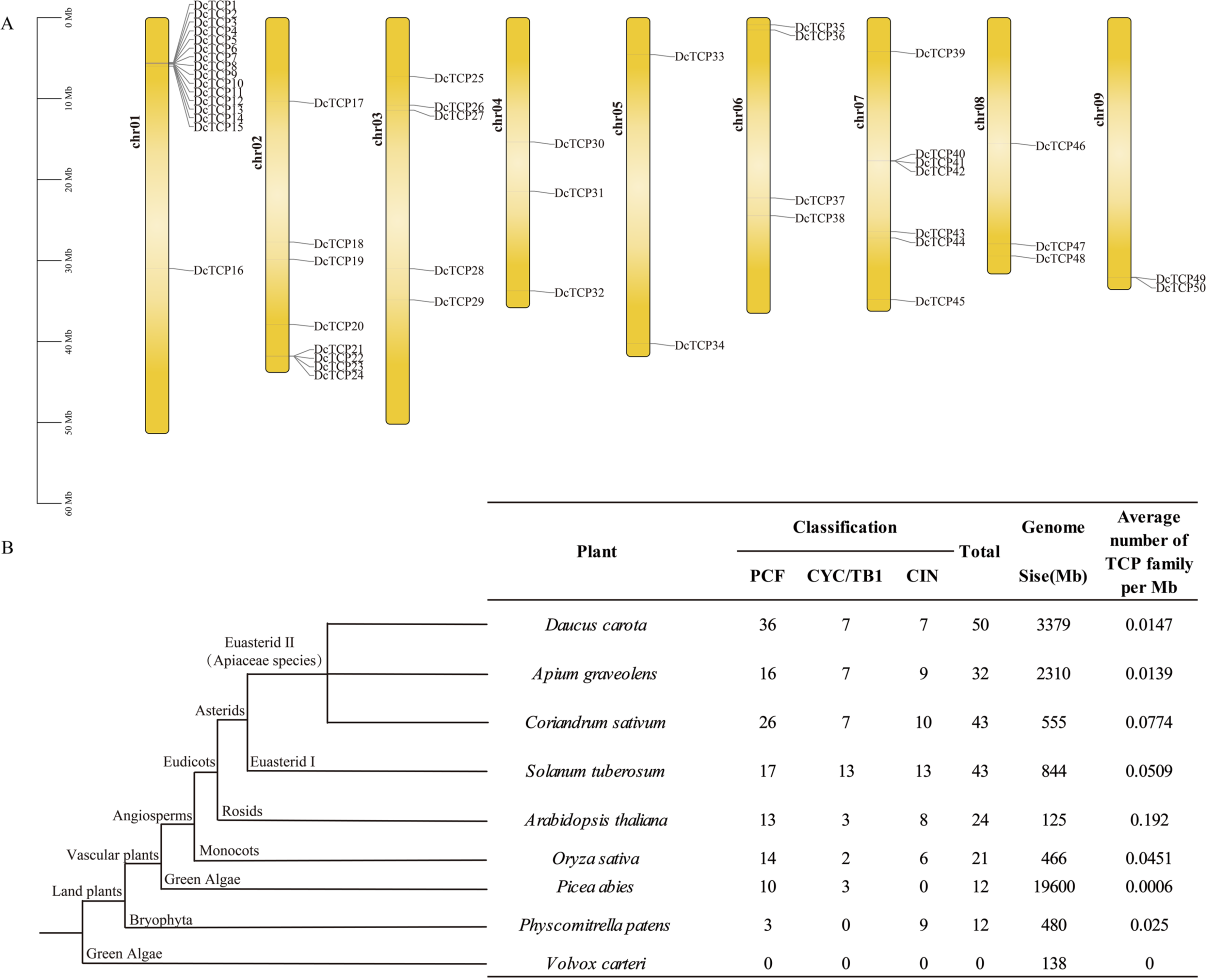
The distribution of the TCP gene on the chromosome and the number of TCP genes in different species. **(A)** Location of DcTCP genes on carrot chromosomes. **(B)** The number of TCP genes in different species.

To further clarify the chromosomal distribution of *DcTCP* genes in carrot, a chromosomal localization map of the *DcTCP* gene family was constructed using the bioinformatics tool TBtools (**Figure 1A**). Analysis results indicated that the 50 *DcTCP* genes were unevenly distributed across all 9 carrot chromosomes, and these genes were designated as *DcTCP1* to *DcTCP50* based on their sequential positions on the chromosomes. Among the 9 chromosomes, chromosome 1 harbored the largest number of *DcTCP* genes (16 in total), whereas chromosomes 5 and 9 contained the fewest, with only 2 *DcTCP* genes each. For duplication event classification, genes located on the same chromosome with an intergenic distance of less than 200 kb were defined as tandem duplicates; all other duplicated gene pairs were classified as segmental duplicates (Cheung et al., 2003). To compare the evolution of *TCPs* among plants, we conducted for *TCP* comparisons of 9 species and constructed schematics of plant evolution (**Figure 1B**). Higher plants have far more members of the transcription factor family than lower plants. The number of transcription factor families varies greatly among different species, the most abundant being PCF, followed by CIN. The carrot *TCP* family has 50 members, and the PCF subfamily has 36 members, accounting for 72% of the family.

**Figure 2:**
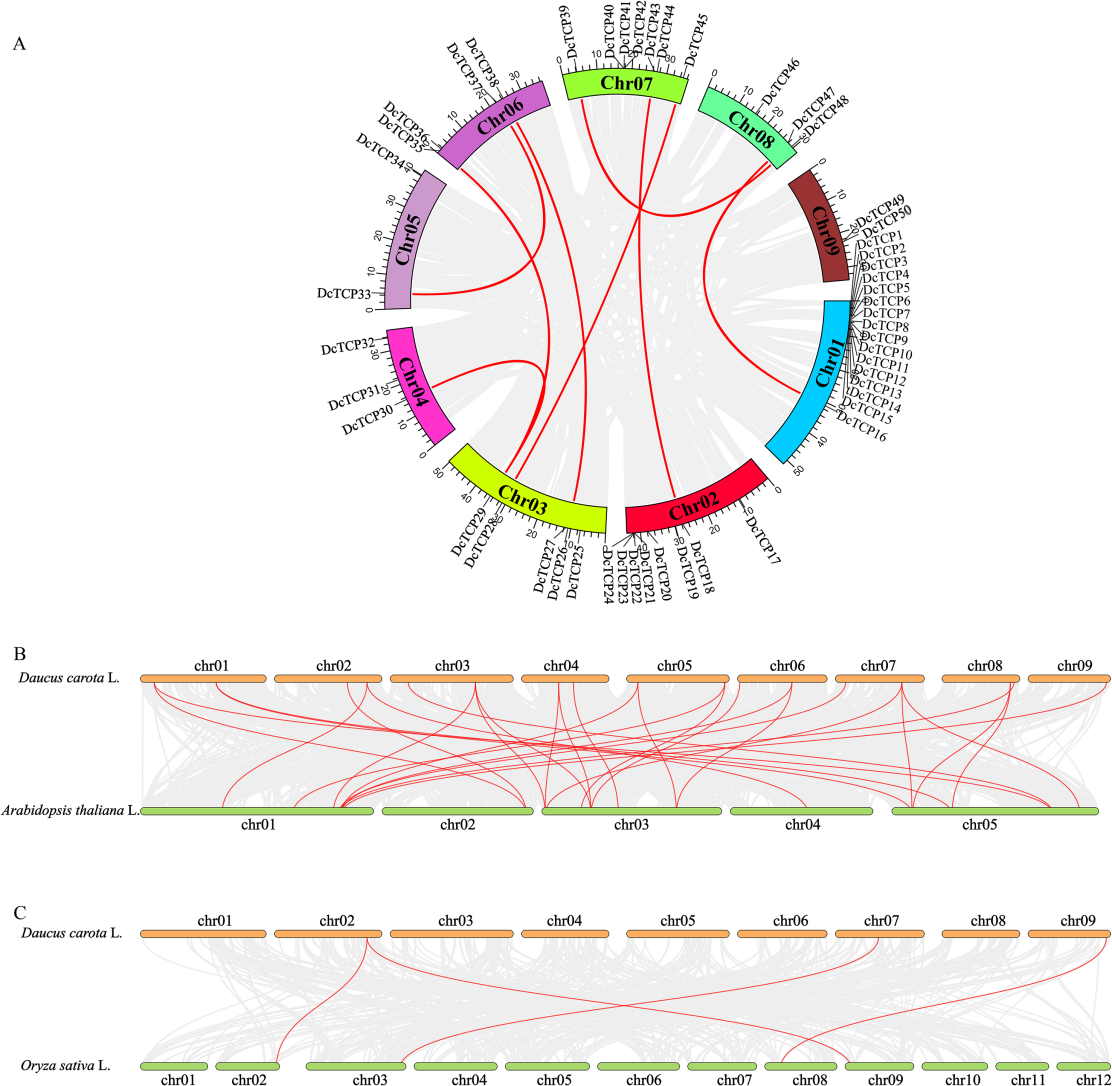
Intraspecific **(A)** and interspecific **(B, C)** collinearity analysis of *TCP* genes. **(A)** The red line indicates segmental duplication. **(B, C)** The red line represents the gene pairs that have a collinearity relationship with carrot and *Arabidopsis thaliana* and rice.

The results revealed that the *DcTCP* gene family contained 22 pairs of tandem duplicated genes and 8 pairs of segmental duplicated genes (**Figure 2A**). Notably, *DcTCP29* formed duplicated pairs with two distinct members, namely *DcTCP30* and *DcTCP36*; all other duplicated genes in the family exhibited a one-to-one pairing pattern. The Ka/Ks of 8 pairs of segmental duplicated genes are less than 1, which were purify selection (**Supplementary Table S3**). Whole-genome duplication (WGD) was the predominant type in carrot (32.0%), These results demonstrate that WGD played an important role in TCP gene expansion in carrot, which is supported by the previous suggestion that they underwent two WGD events since their divergence from lettuce (Song et al., 2020).

Additionally, to clarify the evolutionary relationships of TCP genes, the synteny analysis were performed between carrot and two model plants, Arabidopsis thaliana (**Figure 2B**) and rice (**Figure 2C**). Results showed that carrot shares 29 pairs of syntenic genes with A. thaliana, whereas only 4 pairs of syntenic genes were identified between carrot and rice. This observation indicated that carrot exhibits higher *TCP* gene homology with *A. thaliana* than with rice.

### 3.3 Phylogenetic development of carrot TCP gene family members

**Figure 3:**
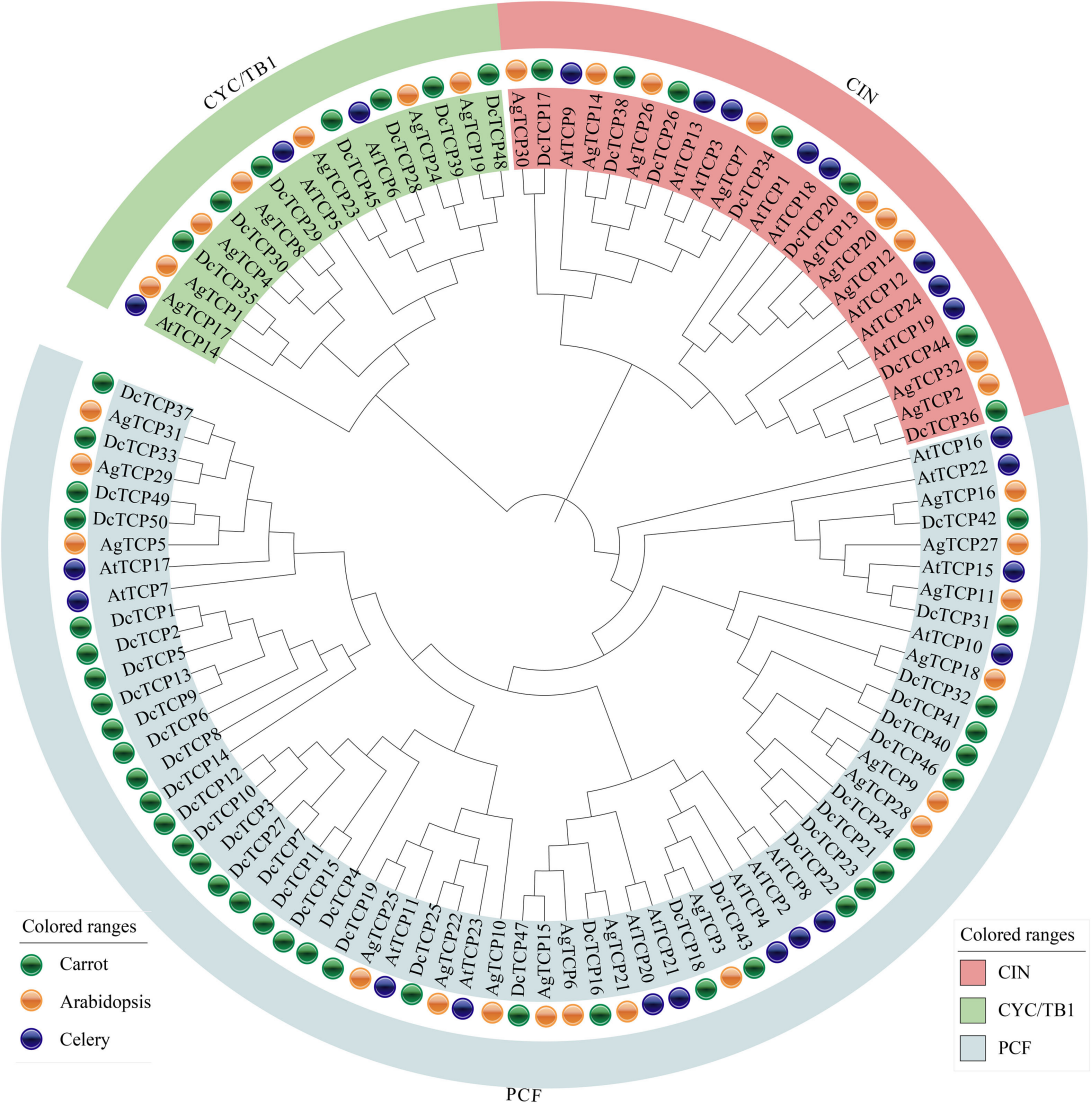
Phylogenetic tree of TCP family members in carrot, celery and *Arabidopsis thaliana*. Different groups and TCP proteins from different plant species were distinguished by different colors. Dc: carrot; Ag: celery; At: *Arabidopsis thaliana*.

To investigate the phylogenetic relationships of *TCP* genes between carrot and rice, a phylogenetic tree was constructed using the maximum likelihood (ML) method. This tree included 50 *DcTCP* genes from carrot, 27 *AgTCP* genes from celery and 24 *AtTCP* in *Arabidopsis thaliana* (**Figure 3**). Phylogenetic analysis revealed that *TCP* genes were clustered into three distinct subfamilies—PCF, CIN, and CYC/TB1—consistent with the subfamily classification of *TCP* genes in other plant species. Among these subfamilies, the PCF subfamily contained the largest number of members, with 36 *DcTCP* genes, 16 *AgTCP* genes and 13 *AtTCP* genes. The CYC/TB1 subfamily had the fewest members, with a total of 17 genes. We identified three TCP genes—namely, *DcTCP34* and *AgTCP2*, —that clustered together with *AtTCP3*, suggesting that they are also related to cotyledon fusion (Koyama et al., 2010). Overall, the TCP family members from carrot, *Arabidopsis thaliana* and celery showed similar evolutionary patterns, suggesting that the plant TCP gene family may have originated from a common ancestral gene and retained conserved evolutionary characteristics during speciation.

### 3.4 Analysis of gene structure and protein domain of carrot TCP gene family

The diversity of exon/intron structures and protein domain architectures played a crucial role in the evolution of gene families. For the 50 *DcTCP* family members identified in carrot, most (47 out of 50) contained only one exon; only *DcTCP45* has two exons, and *DcTCP11* has three exons. None of the *DcTCP* genes harbor introns, indicating a relatively simple gene structure. Further analysis of the protein domains encoded by the *DcTCP* family revealed that all 50 DcTCP proteins contained the canonical TCP domain—a signature feature of the TCP gene family (**Supplementary Figure S1**), confirming their classification as TCP family members.

**Figure 4:**
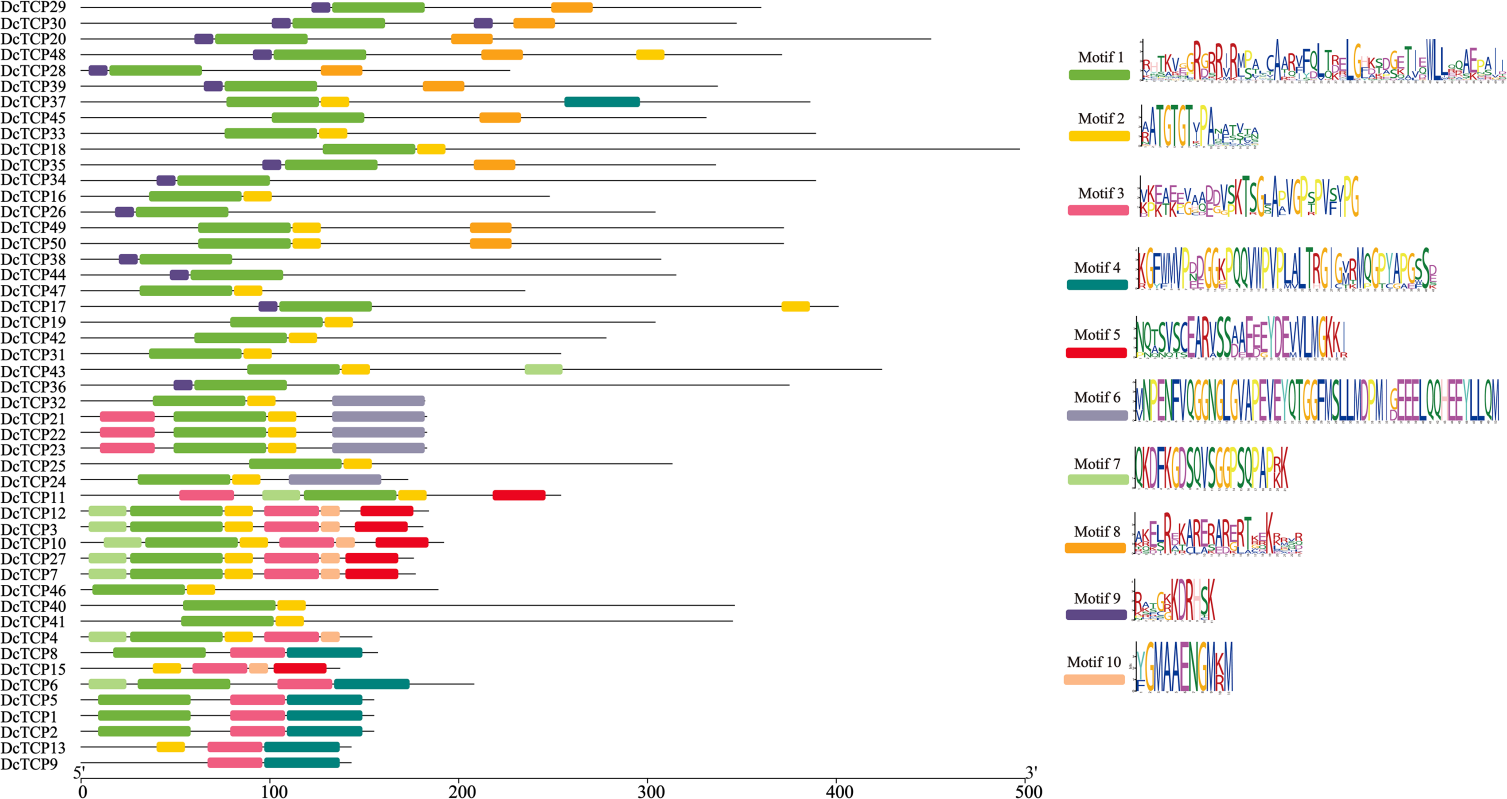
Analysis of the gene structures of the TCP gene family of carrot.

To explore conserved motifs among DcTCP proteins, the MEME (Multiple Em for Motif Elicitation) tool was used to identify 10 conserved motifs (**Figure 4**). Most DcTCP proteins (42 out of 50) contained at least four of these motifs. Among the 10 motifs, Motif 1 was the most widely distributed, presented in all DcTCP proteins except DcTCP9 and DcTCP13. Motif 2 was detected in 31 DcTCP proteins, while Motif 6 was the least abundant, present in only 5 DcTCP proteins. Additionally, spatial analysis showed that most of these conserved motifs were localized within the first 200 amino acid (aa) residues of the DcTCP proteins.

### 3.5 Analysis of cis-elements in the promoter of carrot TCP gene family

**Figure 5:**
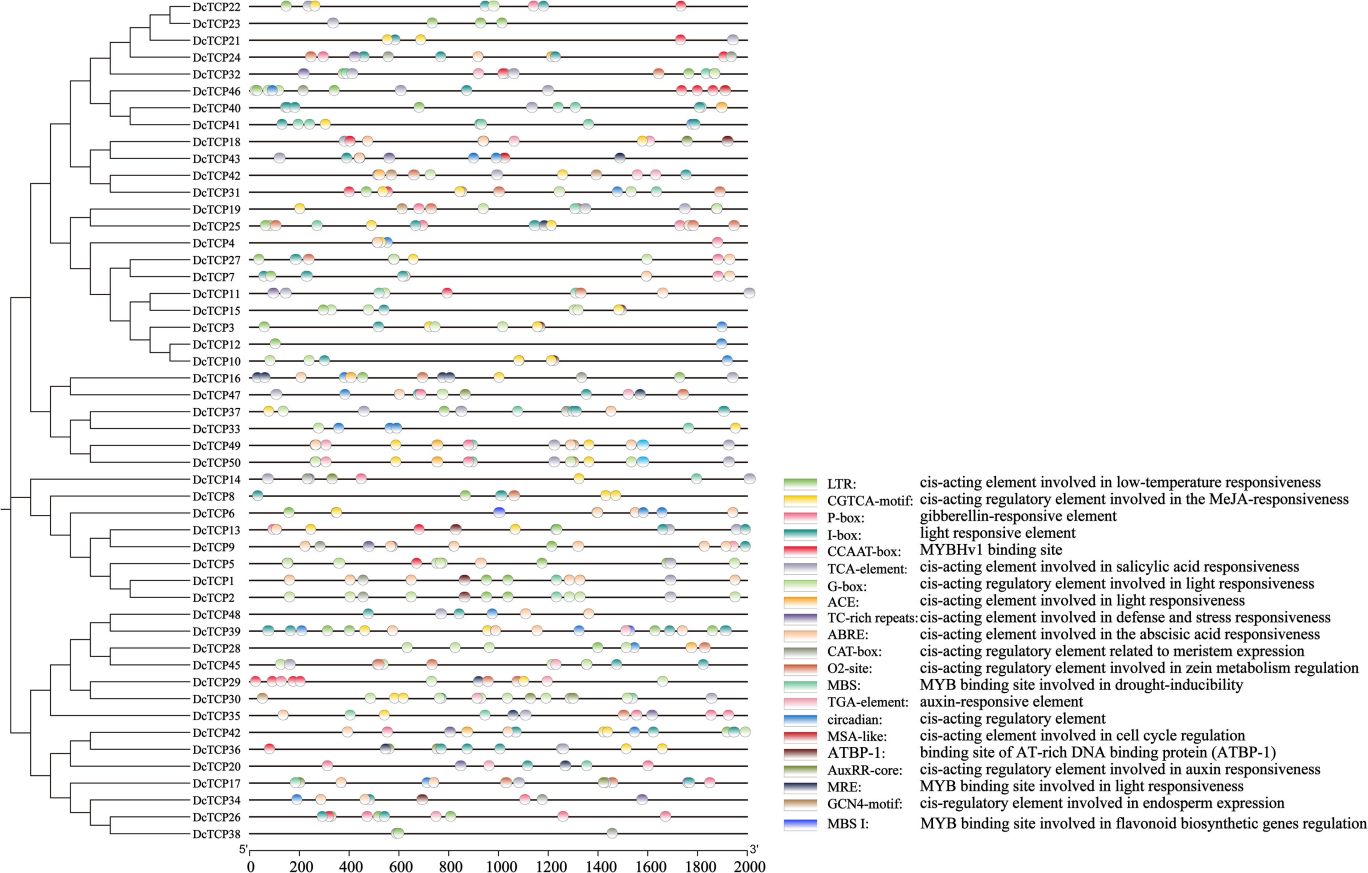
Analysis of cis-type elements of *TCP* gene family promoters of carrot. The cis-elements were represented by distinct colors.

PlantCARE analysis revealed the presence of abundant responsive regulatory elements within the promoter region of the *DcTCP* genes (**Figure 5**). The identified cis-acting elements were categorized into four distinct groups based on their functional annotations. The first group comprised hormone-responsive elements, including but not limited to the CGTCA-motif, P-box, TCA-element, ABRE, TGA-element, and AuxRR-core. The second group consisted of photoresponse-associated elements, such as the I-box, G-box, ACE, and MRE. The third group encompasses growth and development regulatory elements, which included the CAT-box, O2-site, CCAAT box, and circadian element. The fourth group was defined as stress-responsive elements, featuring the ATBP-1, LTR, TC-rich repeats, MBS, and MBS I. Further comparative analysis demonstrated that each DcTCP promoter contained conserved elements, including MYB, P-box, CAAT-Box, and LTR. Notably, promoters of members belonging to the carrot PCF subfamily harbored all the predicted cis-acting elements. In contrast, elements such as GCN4-motif, CCAAT-box, and ACE were not detected in promoters of the CYC/TB1 subfamily. These findings collectively suggested that PCF subfamily members in carrot exhibit more extensive regulatory functions.

### 3.6 GO function analysis of carrot TCP gene family

**Figure 6:**
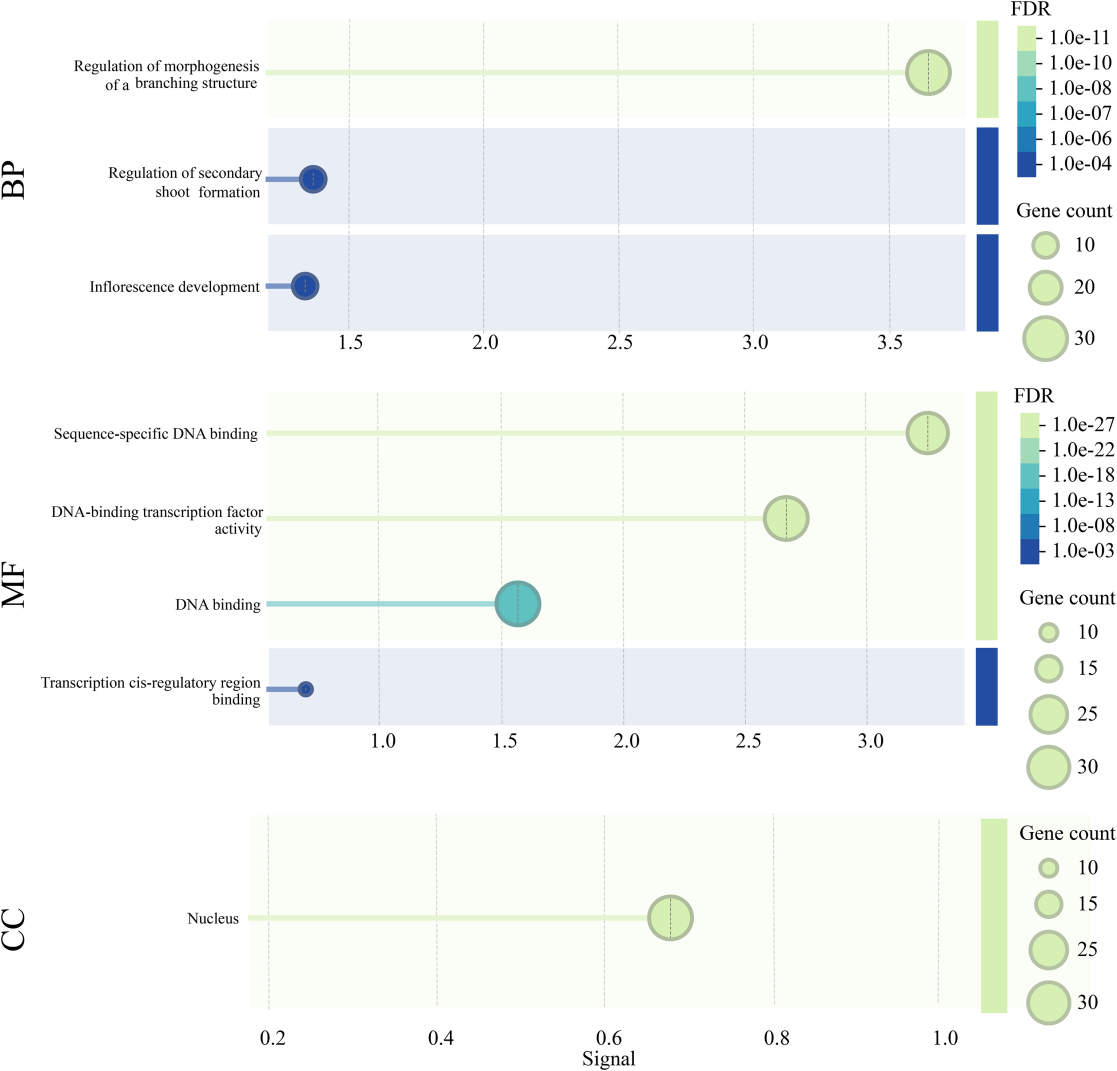
GO function of the TCP gene family of carrot. BP, Biological Process; MF, Molecular Function; CC, Cellular Component.

To more comprehensively delineate the potential functions of TCP genes in carrot, detailed functional annotation and classification were performed using the EggNOG 5.0 tool (**Figure 6**). Functional enrichment analysis revealed that DcTCPs were involved in a broad spectrum of biological processes, including regulation of morphogenesis of a branching structure, Regulation of secondary shoot formation, and inflorescence development. In terms of molecular functions, DcTCPs primarily exhibited binding activity, and transcription factor activity, among others. For cellular component classification, nucleus was identified as the major categories.

### 3.7 Interaction miRNA and protein network analysis of carrot TCP gene family members

**Figure 7:**
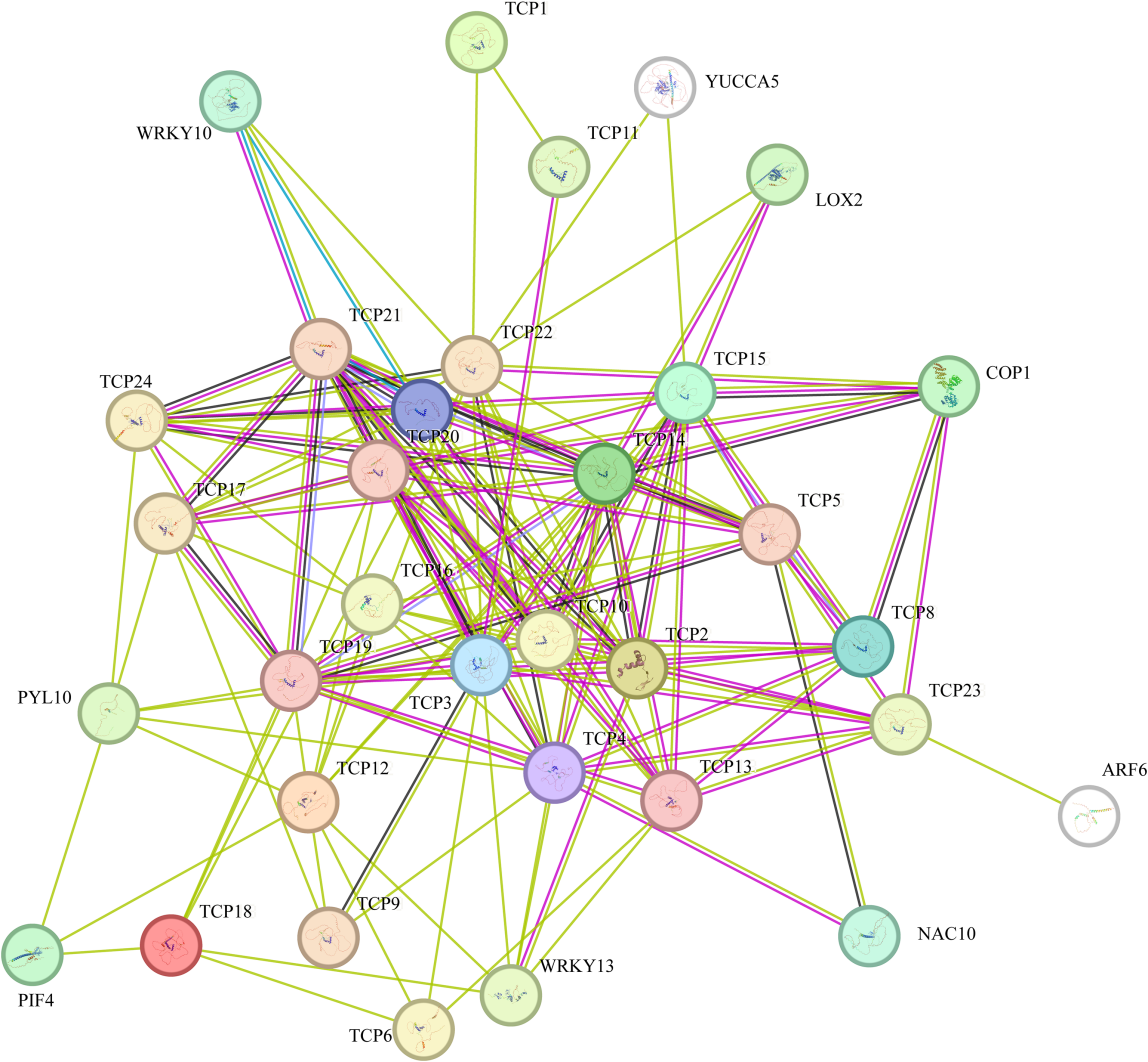
Analysis of *TCP* gene family protein networks in carrot.

As a crucial family of transcription factors, the TCP gene family exerted its core functions primarily through protein-protein interactions. As illustrated in **Figure 7**, protein-protein interaction (PPI) network prediction was performed using *Arabidopsis thaliana* as a reference organism. The results demonstrated that all 28 member proteins of the DcTCP family were capable of interacting with other proteins, leading to the identification of a total of 214 protein pairs. Notably, *DcTCP4*, *DcTCP3*, *DcTCP14*, and *DcTCP10* exhibited the highest number of interacting proteins. cTCP33 and DcTCP37 maintained high expression levels during the entire root development stage (30 d to 95 d), which corresponds to the key period of carrot root hypertrophy and nutrient accumulation; this suggests that these two genes may regulate cell expansion and storage substance accumulation in fleshy roots. Collectively, these findings suggested that these specific DcTCP proteins may participated in diverse biological regulatory mechanisms by recruiting or interacting with a larger repertoire of target proteins.

MicroRNAs (miRNAs) are key regulatory molecules in plants, primarily mediating post-transcriptional gene expression regulation. To identify miRNAs targeting the coding regions of DcTCP genes, prediction analysis was performed using the psRNA Target online tool, with detailed results summarized in the **Table 2** (**Supplementary Table S5**). A total of 12 miRNAs were identified to be involved in the regulation of DcTCP genes. Among these, *miR319* exhibited the highest number of target DcTCP genes (7 targets), followed by *miR172* (6 targets). In contrast, *miR5072* had the fewest targets, regulating only the *DcTCP34* gene. Regarding the mechanism of action, the predicted regulatory mode of most identified miRNAs on their target *DcTCP* genes was direct cleavage. Notably, *miR402* was the only miRNA predicted to exert its regulatory effect through translational inhibition.

**Table 2:** miRNA characteristic information of the TCP gene family interaction in carrot.

miRNA ID	Target gene	Expectation	miRNA aligned fragment	Inhibition way
*miR156*	*TCP9/13/30/31*	5	GCUCACCUCUCUUUCUGUCAGU	Cleavage
*miR157*	*TCP29/49/50*	5	GCUCUCUAUACUUCUGUCACC	Cleavage
*miR159*	*TCP35/38/44/46*	5	AUUGGAGUGAAGGGAGCUCCA	Cleavage
*miR166*	*TCP31/39/40/42*	5	UCGGACCAGGCUUCAUUCCCC	Cleavage
*miR172*	*TCP30/43/46/47/49/50*	5	AGAAUCUUGAUGAUGCUGCAG	Cleavage
*miR319*	*TCP4/5/7/20/26/38/43*	5	UUUGGACUGAAGGGAGCUCCU	Cleavage
*miR396*	*TCP4/7/20/27*	5	UUCCACAGCUUUCUUGAACUU	Cleavage
*miR402*	*TCP49/50*	5	UUGGCCUAUUGAACCUCUGUUU	Translation
*miR408*	*TCP22/23/43/45*	5	CUGCACUGCCUCUUCCCUGGC	Cleavage
*miR2275*	*TCP17/25/32/35*	5	UUUGUUUUUCUCCAAUAUCUCA	Cleavage
*miR828*	*TCP18/20/48*	5	GAGAAGACUUGUUCAAGGAAGA	Cleavage
*miR5072*	*TCP34*	5	CGAUUCCCCAGCGGAGUCGCCA	Cleavage

### 3.8 Expression pattern analysis of carrot TCP gene family members in roots at different stages

**Figure 8:**
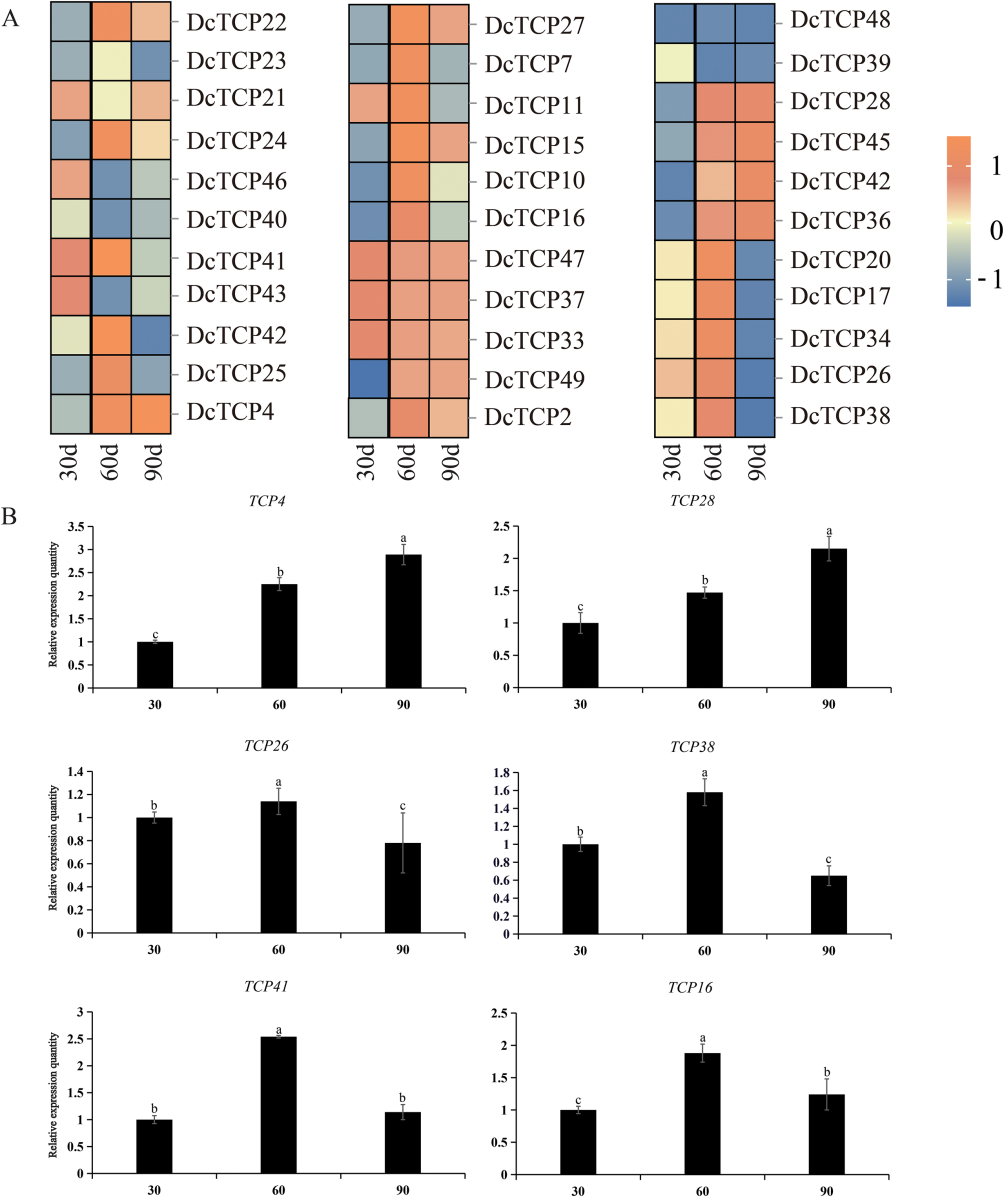
Transcript abundances of *TCP* genes at different developmental stages (30 d, 60 d, and 95 d) in carrot root. **(A)** The expression levels of the TCP gene detected in carrot roots at different periods. **(B)** Partial candidate gene testing by RT-qPCR. Different letters indicate significant differences at the *P* < 0.01 level.

By analyzing and calculating the transcriptome data, the transcriptional abundance of the *DcTCPs* gene in carrot during the development process was obtained. Thirty-three *DcTCPs* gene expressions were discovered. As shown in **Figure 8**, *DcTCPs* were widely expressed at different developmental stages. *DcTCP33*, *DcTCP37* and *DcTCP47* showed relatively high expression levels throughout the entire developmental stage of carrot, while *DcTCP39* and *DcTCP48* exhibited relatively low expression levels throughout the developmental stage. *DcTCP4*, *DcTCP7*, *DcTCP10*, *DcTCP11*, *DcTCP15*, *DcTCP16*, *DcTCP25*, *DcTCP27*, *DcTCP28*, *DcTCP36*, *DcTCP42* and *DcTCP45* gradually increased with the stage of development. We selected six genes for RT-qPCR verification, and the results were consistent with the transcriptome data (**Figure 8B**). It indicated that these genes may be involved in the formation and development of carrot root.

## 4 Discussion

Unlike previous studies that focused on aboveground organ development regulated by TCP genes, our results reveal the specific expression pattern of *DcTCP* genes during carrot root development, which helps to elucidate the molecular mechanism of fleshy root formation in Apiaceae crops. At present, members of the TCP gene family have been identified in multiple species, with 36 existing in soybeans (Feng et al., 2018). With the development of bioinformatics, the number of members in the rice TCP gene family has increased from the initial 22 to 27. The number of TCP members varied among different species, which may be related to genomic replication and chromosomal recombination and translocation (Dujon and Louis, 2017). Multiple studies have established that TCP genes played widespread roles in diverse physiological and biological processes, encompassing plant growth and abiotic stress responses (Liu et al., 2022; Panzade et al., 2024). In this paper, 50 *TCP* transcription factor genes were identified in carrot through bioinformatics methods. Meanwhile, their basic physicochemical properties, gene structures, evolutionary relationships, expression patterns and interactions were analyzed, with the aim of laying a foundation for further in-depth research on the functions of the TCP gene family.

Like most plants, the gene structure of the TCP family in carrot is relatively simple. Genes within the same subfamily have highly similar exons and fewer than three introns, This structural feature has also been found in tea plants (Shang et al., 2022), *Cymbidium goeringii* (Liu et al., 2022), and *Dactylis glomerata* (Wang et al., 2023). Genes with fewer introns can rapidly produce more proteins and respond quickly to abiotic stress (Ma et al., 2020). For the *DcTCP* genes, the deletion of introns may be a strategy in response to abiotic stress. Although the amino acid sizes, molecular weights and isoelectric points of different members vary greatly, almost all carrot DcTCP proteins were located in the nucleus. From this, it can be inferred that the carrot TCP family genes mainly participate in the transcriptional regulation of genes in the nucleus. Conserved motif analysis revealed that all 48 genes contained motif 1, and this motif was highly similar to the TCP domain sequence, similar to the tomato TCP family (Parapunova et al., 2014). The gene structure and conserved motifs of the same subfamily were basically the same, which means that their biological functions are the same or similar. There are significant differences in the gene motifs of different subfamilies, especially the PCF and CIN subfamilies. This has also become an important means to distinguish different subfamilies. Through chromosome localization analysis, it was found that the DcTCP genes are unevenly distributed on the 9 chromosomes of the carrot genome. The uneven distribution of this gene on the genome chromosomes is closely related to the common phenomenon of gene loss in angiosperms (Sun et al., 2023). The PCF subfamily accounted for 72% of the total *DcTCP* genes, which is a higher proportion than that in *Arabidopsis* (54%, 13/24) and celery (50%, 16/32). This expansion may be related to the specific evolutionary adaptation of carrot, especially the development of fleshy roots. Previous studies have shown that PCF subfamily members primarily regulate cell proliferation and elongation in plants; thus, the expansion of the PCF subfamily in carrot may provide abundant genetic resources for the precise regulation of root cell expansion and hypertrophy—a key process in carrot fleshy root formation. This finding suggests that the PCF subfamily may have undergone functional differentiation during carrot evolution to meet the demand for root development.

The *cis*-acting element is a segment of DNA sequence in front of the coding region of a gene and plays a significant role in the expression of gene functions (Huang et al., 2021). The prediction results showed that *DcTCPs* contain multiple functional elements such as light response, growth and development regulation, various stress responses, and hormone responses. Meanwhile, the GO functional analysis also revealed that biological metabolic processes, biological regulation, responses to stimuli, and cellular components were the main functions of the *DcTCPs* gene family members. Each DcTCP promoter contained components such as MYB, P-box, CAAT-Box, LTR, etc., which was consistent with the studies of other crops on the participation of the TCPs gene family in photosynthesis, hormone regulation, growth and development, stress response, etc (Koyama et al., 2010; Ling et al., 2020). The cis element and GO functional analysis further demonstrate that although the TCP gene families of different species have different evolutionary processes, their main functions are similar and highly conserved.

Interaction network analysis revealed that 28 member proteins of the carrot DcTCP family formed a total of 214 protein pairs with other proteins, among which the interacting genes of the same family accounted for the majority. The expression levels of DcTCP4/7/10 gradually increased with root development, consistent with the physiological process of secondary growth in carrot roots, indicating their potential role in vascular tissue differentiation and cell wall thickening. In addition, it interacted with members of the transcription factor family such as WRKY (Hao et al., 2012) and NAC (Gu et al., 2024), and regulated the growth and development process of carrot by interacting with various hormone pathway proteins (Ferrero et al., 2019; Zhou et al., 2019). These interactions may form a regulatory module to coordinate hormone signaling (e.g., auxin and gibberellin) and stress responses, as reported in other plant species. miRNA, as an important regulatory element, plays an irreplaceable role in the growth and development of plants (Lian et al., 2018). A total of 12 miRNAs and carrot TCP family members were predicted to interact in this article. Among them, the TCP genes interacting with *miR319* were the most numerous. *miR319* regulated flower formation, lateral root development and leaf development in various plants by splicing TCP genes and inhibiting their protein expression (Baulies et al., 2022; Nag et al., 2009). Our results suggest that miR319 may play a conserved role in carrot by targeting *DcTCP4/5/7* genes, thereby modulating cell proliferation and elongation during fleshy root development. It is worth noting that *miR159* exhibited the same role as *miR319* during the leaf development of *Arabidopsis thaliana* (Du et al., 2023), which also indicated that different miRNAs synergistically regulate plant growth and development. These research conclusions also provided more references for studying the regulatory mechanism of miRNA on carrot.

During the evolution of plants from lower to higher levels, a multi-level regulatory mechanism covering growth and development regulation, metabolic network balance, and response to adverse stress has gradually been formed to adapt to complex living environments and precise regulatory requirements. The rapid expansion of the TCP transcription factor family was regarded as one of the important evolutionary strategies for plants to optimize gene resources to meet the demands of the above-mentioned multiple regulatory pathways. The functional differentiation and synergy of its family members may provide a key molecular basis for the adaptive evolution of plants. The expression patterns of the TCP gene family members in carrot showed significant developmental period-dependent dynamic change characteristics. This spatiotemporal specific expression pattern suggested that different *DcTCP* genes may exercise their biological functions through differentiated regulatory pathways (such as spatiotemporal specific expression, protein-protein interaction or target gene selection, etc.), and thereby jointly participate in the precise regulation of the growth and development process of carrot. It is worth noting that some carrot *DcTCP* members (such as *DcTCP33*, *DcTCP37* and *DcTCP47*) exhibited a high degree of consistency in key features such as expression patterns (such as tissue specificity and expression trends at developmental stages) and gene structures (such as exon-intron composition and conserved domain distribution). This suggested that there may be a phenomenon of functional redundancy in this subgroup of genes. The existence of such functional redundancy provided a guarantee for the robustness of key biological processes in plants, and also offered new research directions and experimental design ideas for the subsequent analysis of the core functional modules of the *DcTCP* family through technologies such as gene editing and gene silencing, as well as the screening of key gene loci with breeding application value, accelerate the application of these findings in crop improvement and promote the breeding of stress-resistant and high-yield carrot varieties.

## 5 Conclusion

This study systematically identified and analyzed the TCP transcription factor family in carrot (*Daucus carota* L.) using bioinformatics tools, combined with transcriptomic and RT-qPCR validation. A total of 50 *DcTCP* genes were identified, unevenly distributed across 9 chromosomes and clustered into PCF, CIN, and CYC/TB1 subfamilies, with the PCF subfamily (36 members, 72%) dominating. DcTCP proteins are predominantly hydrophilic, unstable, and nuclear-localized, with simple gene structures (47 containing one exon) and conserved TCP domain (motif 1). Whole-genome duplication (WGD) drove family expansion, and carrot showed closer TCP gene homology with *Arabidopsis* than rice. Cis-acting elements and GO analysis indicated DcTCP involvement in light response, hormone signaling, stress tolerance, and development. Twelve miRNAs (*miR319* targeting 7 genes) and 214 protein-protein interaction pairs were predicted, revealing complex regulatory networks. Expression patterns showed *DcTCP33/37/47* were highly expressed throughout root development, while *DcTCP4/7/10* et al. exhibited increasing expression, confirming roles in fleshy root formation.

This study enriches plant TCP research, provides a theoretical basis for carrot development studies, and offers valuable gene resources for breeding high-quality, stress-resistant varieties.

## Data Availability

The original contributions presented in the study are included in the article/**Supplementary Material**. Further inquiries can be directed to the corresponding author/s.
