# Supplementary material for: Genome-wide identification, classification, and expression pattern analysis of the TCP transcription factor family in carrot
Source: Front Plant Sci. 2026 Feb 26;17:1788962. doi: 10.3389/fpls.2026.1788962 (PMC12980029; doi:10.3389/fpls.2026.1788962)
Supplement: Supplementary file 2 [file Table2.docx]

Supplementary Material

# Supplementary Data

Supplementary Material should be uploaded separately on submission. Please include any supplementary data, figures and/or tables.

Supplementary material is not typeset so please ensure that all information is clearly presented, the appropriate caption is included in the file and not in the manuscript, and that the style conforms to the rest of the article.

# Supplementary Figures and Tables

For more information on Supplementary Material and for details on the different file types accepted, please see [here](https://www.frontiersin.org/guidelines/author-guidelines#supplementary-material).

## Supplementary Figures





**Supplementary Figure 1.** TCP gene family domain in carrot.
